# Effects of Prohydrojasmon on the Number of Infesting Herbivores and Biomass of Field-Grown Japanese Radish Plants

**DOI:** 10.3389/fpls.2021.695701

**Published:** 2021-08-12

**Authors:** Kengo Yoshida, Masayoshi Uefune, Rika Ozawa, Hiroshi Abe, Yuka Okemoto, Kinuyo Yoneya, Junji Takabayashi

**Affiliations:** ^1^Faculty of Agriculture, Meijo University, Nagoya, Japan; ^2^Center for Ecological Research, Kyoto University, Otsu, Japan; ^3^RIKEN BioResource Research Center, Tsukuba, Japan; ^4^Graduate School of Agriculture, Meijo University, Nagoya, Japan; ^5^Faculty of Agriculture, Kindai University, Nara, Japan

**Keywords:** prohydrojasmon, *Raphanus sativus* L. var. *hortensis* Backer, aphids, parasitoids, thrips, vegetable weevils, leaf-mining fly larvae, lepidopteran larvae

## Abstract

Prohydrojasmon (PDJ), an analog of jasmonic acid (JA), was found to induce direct and indirect defenses against herbivores in non-infested plants. To test whether PDJ can be used for pest control in crop production, we conducted experiments in pesticide-free Japanese radish fields from October 4 to December 12 in 2015. Twenty-four Japanese radish plants in three plots were treated with a 100 times-diluted commercial formulation (5%) of PDJ (treated plants), and 24 plants in three different plots were treated with water (control plants) until November 29 every week. Throughout the observation period, the number of aphids, leaf-mining fly larvae, vegetable weevils, and thrips was significantly lower on the treated plants than on the control plants. In contrast, the number of lepidopteran larvae was not significantly different between the treated and control plants throughout the study period. Parasitized aphids (mummies) were also observed in both plots. Poisson regression analyses showed that a significantly higher number of mummies was recorded on the treated plants as compared to that on the control plants when the number of aphids increased. This suggested that PDJ application to Japanese radish plants attracted more parasitoid wasps on the treated plants than on the control plants. We also identified eight terpenoids and methyl salicylate as the PDJ-induced plant volatiles in the headspace of the treated plants. Some of these volatiles might be responsible for attracting aphid-parasitoid wasps in the field. However, for other insect pests, we did not find any natural enemies. Interestingly, the genes of the JA and salicylic acid signaling pathways were differentially upregulated in the treated plants. We also observed that the PDJ treatments induced the expression of the genes related to glucosinolate biosynthesis and the subsequent isothiocyanate formation. Additionally, the weights of both the aboveground and belowground parts of the treated plants were significantly lower than those of the respective parts of the control plants. These results indicated that the treatment of Japanese radish plants with a 100 times-diluted commercial formulation of PDJ induced their direct and indirect defenses against several insect pest species to reduce their numbers, and negatively affected their biomass.

## Introduction

In response to herbivory, plants become more resistant either directly (by decreasing their palatability to herbivores) or indirectly (by increasing the effectiveness of carnivorous natural enemies of herbivores). Jasmonic acid (JA), a plant hormone, is involved in the induction of direct defenses against herbivores in infested plants (Smith et al., 2009), and is also involved in the production of herbivory-induced plant volatiles, which attract the natural enemies of the infesting herbivores (Takabayashi and Dicke, 1996; Arimura et al., 2009; McCormick et al., 2012; Takabayashi and Shiojiri, 2019). Exogenous application of JA to non-infested plants can induce direct defenses, making them less suitable resources for herbivores (Farmer and Ryan, 1992; Thaler et al., 1996; Heijari et al., 2005; Cooper and Rieske, 2008). Furthermore, JA-treated non-infested plants become more attractive to predators and parasitoids than untreated plants (indirect defense induction) (Hopke et al., 1994; Dicke et al., 1999; Gols et al., 1999; Thaler, 1999; Ozawa et al., 2000, 2004, 2008; Lou et al., 2005).

Prohydrojasmon (PDJ) [propyl(1*RS*,2*RS*)-(3-oxo-2-pentylcyclopentyl) acetate], an analog of JA, was first registered as a plant growth regulator, particularly for coloring apples and grapes (Koshiyama et al., 2006). Subsequently, similar to JA, PDJ was found to induce direct and indirect defenses against herbivores in non-infested plants (Mandour et al., 2013; Uefune et al., 2014; Sobhy et al., 2015; Matsuura et al., 2020). A report on PDJ treatment of non-infested maize plants suggests a reduction in the weights and survival rates of the common armyworm, *Mythimna separata* (Walker), larvae and their increased attraction to *Cotesia kariyai* Watanabe, a parasitoid wasp of the herbivore (Mandour et al., 2013). Furthermore, Uefune et al. (2014) reported that two-spotted spider mites (*Tetranychus urticae* Koch) laid significantly fewer eggs on PDJ-treated leaf disks than on control leaf disks. They also showed that PDJ treatment of non-infested lima bean plants induced the production of volatiles, including (*E*)-β-ocimene and (*E*)-4,8-dimethyl-1,3,7-nonatriene (DMNT), which attract *Phytoseiulus persimilis* Athias-Henriot, a predatory mite (Dicke et al., 1990). Moreover, under greenhouse conditions, PDJ treatment of tomato plants negatively affects the damage caused by thrips (Matsuura et al., 2020). These laboratory and greenhouse studies indicate that PDJ treatment of crops increases their direct and indirect defenses against several insect pests under open field conditions.

Therefore, in the present study, we investigated whether the treatment of field-grown Japanese radish plants (*Raphanus sativus* L. var. *hortensis* Backer) with commercially formulated PDJ could reduce the number of insect pests throughout the cultivation season and affect the biomass of PDJ-treated Japanese radish plants after harvesting. We also analyzed the volatiles emitted from, and the genes expressed in the treated and control plants. The possible use of PDJ for pest management was discussed.

## Materials and Methods

### Prohydrojasmon

We used a 100 times-diluted commercial formulation with 5% PDJ (Jasmonate-Ekizai^®^, Meiji Seika Pharma, Tokyo, Japan) for the experiments. This concentration was determined based on the results of our previous studies, wherein the induction of direct and indirect defense responses was detected at this concentration in corn (Mandour et al., 2013) and lima bean plants (Uefune et al., 2014). As a plant growth regulator and for controlling thrips, 1000- to 2,000-fold dilution and 500-fold dilution are suggested by the company, respectively.

### Experiments in a Common Garden

We used an open common garden (ca. 3 m × 6 m) located in Gifu City, Gifu Prefecture, Japan for the field experiments in 2015. The common garden was divided into 12 plots (140 cm × 60 cm). We sowed three seeds in each spot (8 spots in two lines per plot: Supplementary Figure 1) on September 27. Seedlings were recognized a week after seeding (that is, October 4), and then the seedlings in each spot were reduced to one (i.e., eight seedling pre plot). Three each of treatment (PDJ-treated) and control (water-treated) plots were randomly chosen using the “RAND” function in Microsoft Excel (Supplementary Figure 1). The observation was performed on October 4, 11, 18, and 25; November 1, 15, 22, and 29; December 6, 13. On October 4, 11, 18, and 25; November 1, 11, 22, and 29, we sprayed the PDJ solution. The 24 Japanese radish plants in the three treatment plots were sprayed with 3 mL of the 100 times-diluted commercial formulation (5%) of PDJ (treated plants), and the 24 plants in the control plots were sprayed with water (control plants) using a hand sprayer. In December, we did not spray PDJ (the land owner’s request). The plants were harvested on December 20. The weather conditions in the experimental period are shown in Supplementary Table 1.

We counted the number of arthropods on the plants, including insect pests and their carnivorous natural enemies, in each plot from October 4 to December 13. The insect pests observed in the plots were aphids, thrips, vegetable weevils (*Listroderes obliquus* Klug), leaf-mining fly larvae, and lepidopteran larvae. The major lepidopteran species were the cabbage butterfly (*Pieris rapae* L.) that is a crucifer specialist, and few species of the subfamilies Hadeninae and Plusiinae those are not crucifer specialists. We also observed aphids parasitized by wasps (mummies). However, the species of aphids, aphid parasitoid wasps, leaf- mining flies, and thrips were not identified.

During the first five observation weeks, we observed herbivorous insects on all eight plants in each plot. We randomly chose six and four plants per plot for the sixth-to-eighth and the ninth weeks, respectively, to observe the herbivorous insects.

After the harvest on December 20, we separated the aboveground and belowground parts of all the harvested plants from the treated and control plots, and weighed the parts to assess the effects of PDJ treatment on the growth of the plants.

### Analyses of Headspace Volatiles of Japanese Radish Plants

To test whether PDJ treatment resulted in the induction of volatiles in Japanese radish plants, we cultivated the plants and collected the headspace volatiles in the laboratory. Between 24 and 27 days after seeding, the PDJ- and water-sprayed plants were placed in a growth chamber at 25 ± 1°C, with an RH of 60 ± 22%, and a 16L:8D light regimen for 24 h. Headspace volatiles from 25- to 28-day-old PDJ-treated and control Japanese radish plants were collected using a Twister (a 10-mm long magnetic stir bar coated with a 0.5-mm thick film of polydimethylsiloxane) (Gerstel Twister^®^, Gerstel GmbH and Co., KG, Germany). A potted plant and 0.1 μg of *n*-tridecane infiltrated into a piece of filter paper (1 cm^2^) were placed in a 2 L glass flask as an internal standard. Two stir bars were then placed inside the glass flask using magnetic rods for 2 h, and volatile collection was replicated five times.

Headspace volatiles collected using the stir bars were analyzed by gas chromatography-mass spectrometry (GC-MS; GC: Agilent 6890; MS: Agilent 5973) with an HP-5MS capillary column (Agilent Technologies Inc., United States) equipped with a thermo-desorption system, cooled injection, and cold trap (Gerstel GmbH and Co., KG, Germany). The GC oven temperature was programmed to increase from 40°C (9 min-hold) to 280°C at 10°C/min. The compounds were tentatively identified using the Wiley database and their mass-spectra and retention times were then verified with those of authentic compounds, except for 4-methylthio-3-butenyl isothiocyanate, α-copaene, germacrene D, and caryophyllene oxide. (*Z*)-3-Hexen-1-yl acetate and (*E*)-β-caryophyllene were purchased from Tokyo Chemical Industry Co., Ltd. (Tokyo, Japan). Myrcene, menthol, and (*E,E*)-α-farnesene were purchased from FUJIFILM Wako Chemical Co., Ltd. (Osaka, Japan); α-Humulene was purchased from Sigma-Aldrich LLC. (St. Louis, MO, United States). DMNT was synthesized in our laboratory. The ion intensity of each peak was normalized by dividing the ion intensity of each peak by that of the internal standard (*n*-tridecane) and plant weights (g). The normalized data were referred to as “relative peak areas.”

### qRT-PCR Analyses

To test whether PDJ-treatment resulted in the induction of defense-related genes in Japanese radish plants, we cultivated the plants and conducted qRT-PCR analyses in the laboratory. Total RNA was extracted from Japanese radish leaves (200 mg FW) using TRIzol reagent (Invitrogen, Carlsbad, CA, United States), and the DNA was degraded using an AccuRT Genomic DNA Removal kit (Applied Biological Materials, Richmond, BC, Canada). cDNA was then synthesized from the total RNA (4 μg) using the Thermoscript RT-PCR kit (Invitrogen, Carlsbad, CA, United States) with oligo(dT)_20_ as a primer, according to the manufacturer’s instructions. Subsequently, qRT-PCR was performed using Power SYBR Green Master mix (Thermo Fisher Scientific, Waltham, MA, United States) on an Applied Biosystems 7500 Real-Time PCR System (Thermo Fisher Scientific, Waltham, MA, United States). The primers used are shown in the Supplementary Table 2.

### Statistical Analyses

We analyzed the effects of the PDJ treatment, observation week, and their interaction on the number of aphids, mummies, thrips, and vegetable weevils and the leaf-mining fly larvae, *P. rapae*, and species of the subfamilies Hadeninae and Plusiinae on the radish plants. For the analyses, we used generalized linear mixed models (GLMMs) with Poisson distribution using the “glmer” function in the “lme4” package version 1.1-21 (Bates et al., 2015) in R version 3.3.5 (R Core Team, 2019). The identification of plants was a random effect in all the models, and the significant values from the GLMMs were calculated by the type II Wald chi-square tests using the “Anova” function in the “car” package version 3.0.2 (Fox and Weisberg, 2011) in R.

When the interaction significantly affected the number of insects, we analyzed the effect of treatment on the number in each observation week using a GLMM with Poisson distribution using the “glmer” function in the “lme4” package in R. Additionally, the observation weeks with no target insect in any of the plots were excluded from the analysis. The plots were included as random effects in all the models, and the significant values from the GLMMs were calculated by the likelihood ratio tests using the “anova” function in R. In the event of convergence errors, the models were fitted using the “bobyqa” optimizer in R.

The effect of the treatment on the weights of the aboveground and belowground parts of the plants was analyzed using a GLMM with Gaussian distribution using the “lmer” function in the “lme4” package in R. The plots were included as random effects in all the models, and the significant values from the GLMMs were calculated using the likelihood ratio tests and the “anova” function in R.

The emitted volatiles and the expression of genes in the radish plants were analyzed using *t*-tests in JMP version 14.2.0 (SAS Institute, 2018). The data were Box-Cox transformed in JMP before the analyses. When a dataset had 0 values, 1 was add to all values in the dataset before Box-Cox transformation.

To clarify whether the structure of insect community and the blend of volatile compounds differed between treated plants and control plants in October, November and December, we conducted principal coordinates analysis (PCoA) and a permutational multivariate analysis of variance (PERMANOVA; number of permutations performed = 9999) based on the Bray-Curtis dissimilarities of the number of insects or the relative peak area of volatile compounds of the Japanese radish plants. PCoA and PERMANOVA were calculated using the functions “capscale” and “adonis” in the “vegan” package (Oksanen et al., 2020) in R. Before PERMANOVA was conducted, we used the “betadisper” function of PERMDISP to evaluate whether the dispersion of the insect community structure and volatile compound composition of treated plants differed significantly from those of control plants. Only when no significant difference in the dispersion is observed does a significant difference determined by the PERMANOVA indicate a difference in the insect community structure and the volatile compound composition. We also identified major insects and volatile compounds that are responsible for the differences between treatments via SIMPER analysis using the function “simper.” Before PCoA, PERMANOVA, and PERMDISP were conducted, the number of aphids and mummies was square root transformed. The data on the radish plants with no insects were removed from the analysis using PCoA and PERMANOVA. For graphics, we used the “ggplot2” package (Wickham, 2016).

## Results

### Herbivorous Arthropods Found on Japanese Radish Plants

**FIGURE 1:**
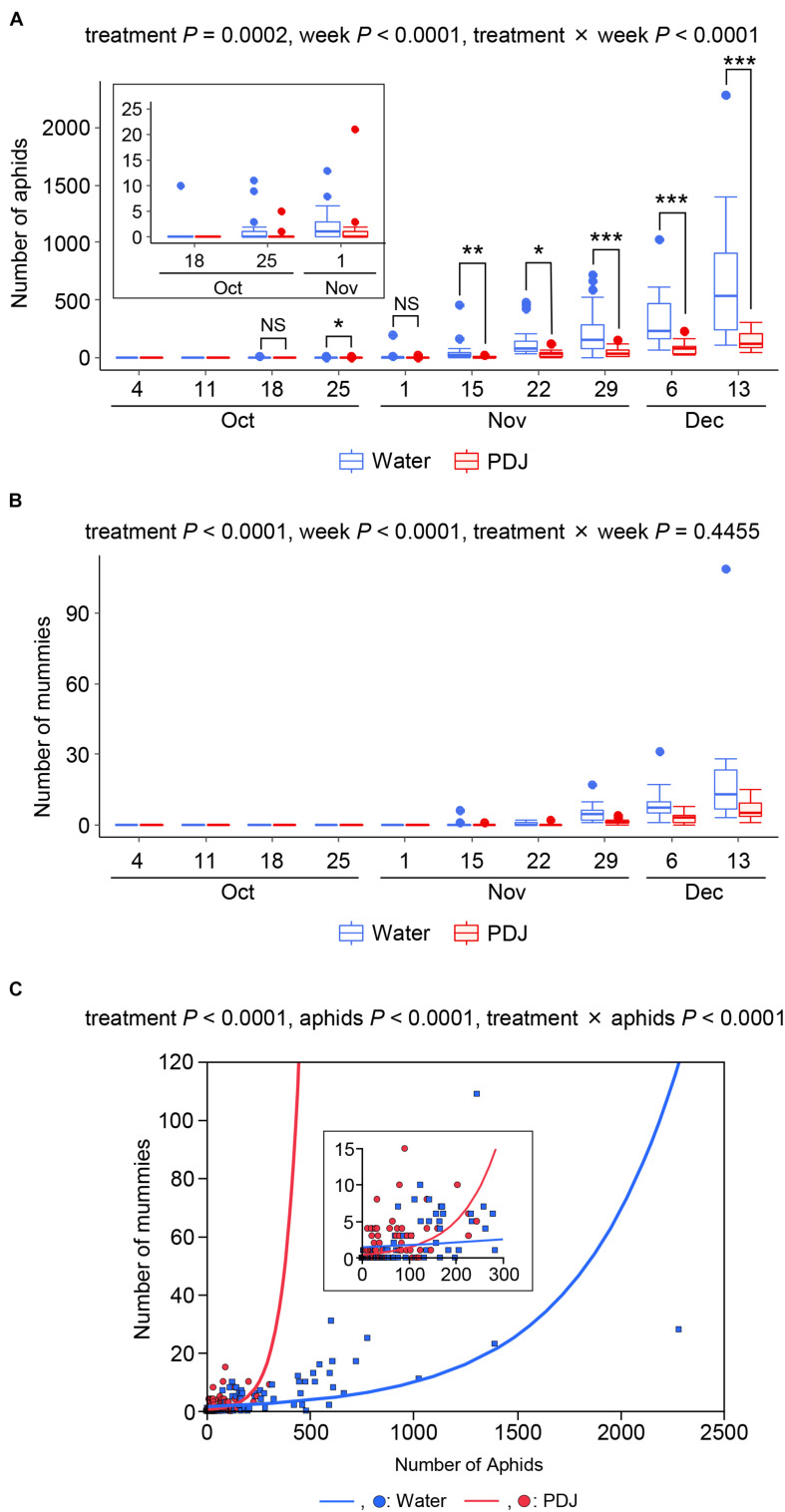
Occurrences of aphids **(A)** and mummies **(B)** on Japanese radish plants in the field during the observation period. **(C)** Poisson regression analysis of the relationship between the numbers of mummies and that of the aphids. NS: 0.05 < *P*, *0.01 < *P* < 0.05, **0.001 < *P* < 0.01, and ***P < 0.001 (GLMM).

Aphids were observed on both the treated and control plants from October 18 to December 13, with a gradual increase in their number during the observation period (Figure 1A). The effects of treatment, observation week, and their interaction (treatment × week) on the number of aphids were significant (treatment: *P* = 0.0002, week: *P* < 0.0001, and treatment × week: *P* < 0.0001) (Figure 1A). Therefore, we compared the number of aphids in the treatment and control plots on each observation week using GLMM. Except for October 18 and November 1, the incidence of aphids in the treatment plots was significantly lower than that in the control plots.

Mummies (aphids parasitized by wasps) were also observed on both the treated and control plants from November 15 to December 13, with a gradual increase in their number (Figure 1B). The effects of the treatment and the observation week were significant (treatment: *P* < 0.0001 and week: *P* < 0.0001), while the effect of their interaction was not significant (*P* = 0.45) (Figure 1B). These results indicated that the number of wasps was always lower in the treatment plots than in the control plots.

We then analyzed the relationship between the number of aphids and that of mummies in the treatment and control plots using Poisson regression analyses (Figure 1C). The effects of the treatment, number of aphids, and the interaction between treatment and the number of aphids on the number of mummies were significantly different (treatment: *P* < 0.0001, number of aphids: *P* < 0.0001, and treatment × number of aphids: *P* < 0.0001).

**FIGURE 2:**
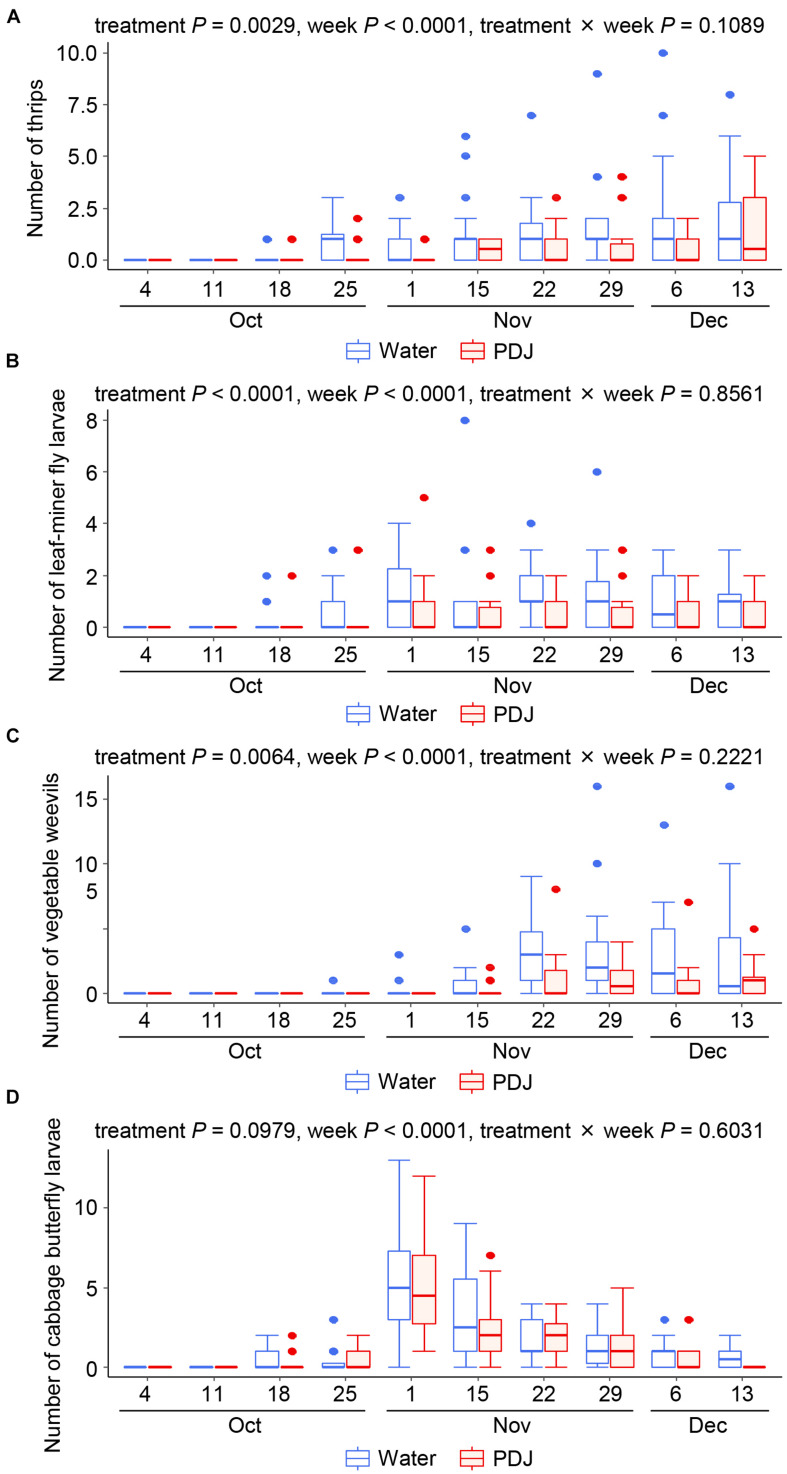
Occurrences of thrips **(A)**, leaf-mining fly larvae **(B)**, vegetable weevils **(C)**, and cabbage butterfly larvae **(D)** on Japanese radish plants in the field during the observation period (GLMM).

Thrips, leaf-mining fly larvae, and vegetable weevils gradually increased on both the treated and control plants from October 18 (October 25 for vegetable weevils) to December 13 (Figures 2A–C). The effects of the treatment (*P* = 0.0029 for thrips, *P* < 0.0001 for leaf-mining fly larvae, and *P* = 0.0064 for vegetable weevil) and the observation week (*P* < 0.0001 for thrips, *P* < 0.0001 for leaf-mining fly larvae, and *P* < 0.0001 for vegetable weevil) on their number were significant, while that of their interaction was not (*P* = 0.11, *P* = 0.86, and *P* = 0.22 for thrips, leaf-mining fly larvae, and vegetable weevil, respectively), indicating that their incidences were always significantly lower in the treatment plots than those in the control plots (Figures 2A–C).

The incidences of the numbers of Lepidopteran larvae of the subfamilies Hadeninae and Plusiinae were quite low (Supplementary Figure 2). The larvae of the cabbage butterfly and species of the subfamily Hadeninae were observed in both the treatment and control plots from October 18 to December 13 (Figure 2D and Supplementary Figure 2A). The effects of treatment (*P* = 0.098 for cabbage butterfly larvae and *P* = 0.63 for Hadeninae larvae) and the interaction between the treatment and the observation week (*P* = 0.60 for cabbage butterfly larvae and *P* = 0.87 for Hadeninae larvae) on the number of larvae were not significant, while that of the observation week was significant (*P* < 0.0001 for the larvae of both the cabbage butterfly and Hadeninae species), indicating that their numbers were not significantly different between the treatment plots and the control plots, but fluctuated significantly during the observation period.

Larvae of the subfamily Plusiinae were observed in the treatment and the control plots from October 4 to December 13 (Supplementary Figure 2B). The number of larvae did not differ significantly between the treatment, the observation week, and the treatment × week interaction (treatment: *P* = 0.95, week: *P* = 0.16, and treatment × week: *P* = 0.26), indicating that the number of larvae was not significantly different between the treatment and control plots without significant fluctuations in their numbers during the observation period.

**FIGURE 3:**
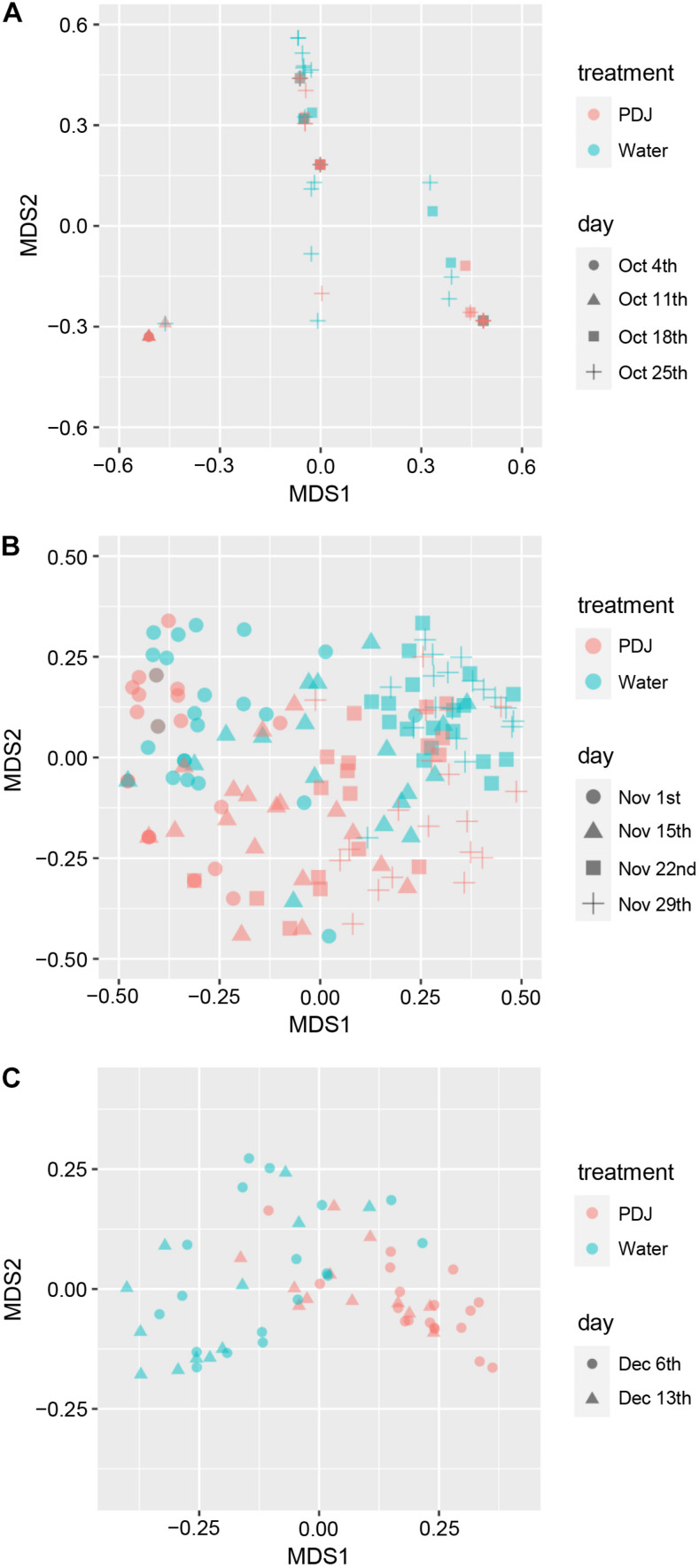
Multidimensional scaling plot (for the first two axes) using a principal coordinates analysis (PCoA) of a Bray–Curtis distance matrix on the data of insect community composition on plants treated with water and those treated with prohydrojasmon (PDJ) in October **(A)**, November **(B)**, and December **(C)**.

To detect compositional differences, we applied a PCoA to the insect communities on the plants treated with water and those treated with PDJ. The first two axes (MDS1 and MDS2) explained the difference (29.5% and 24.8% in October, 33.7% and 13.4% in November, and 42.4% and 12.5% in December) in composition of the insect community between plants. We found that in November and December they were significantly different (PERMDISP: November, *P* = 0.72; December, *P* = 0.27, PERMANOVA: November, *P* = 0.0001; December, *P* = 0.0001, Figures 3B,C) but in October they were not (PERMDISP: *P* = 0.61, PERMANOVA: *P* = 0.12, Figure 3A). The insect species that significantly contributed to the difference between the two groups were aphids (40.1%, *P* = 0.0002), vegetable weevils (10.8%, *P* = 0.0002), leaf mining flies (9.8%, *P* = 0.0002), and thrips (8.1%, *P* = 0.0002) in November and aphids (52.7%, *P* = 0.0001), vegetable weevils (15.9%, *P* = 0.0009), mummies (8.3%, *P* = 0.0016), and cabbage butterflies (4.2%, *P* = 0.028) in December.

### Biomass of the Harvested Japanese Radish Plants

**FIGURE 4:**
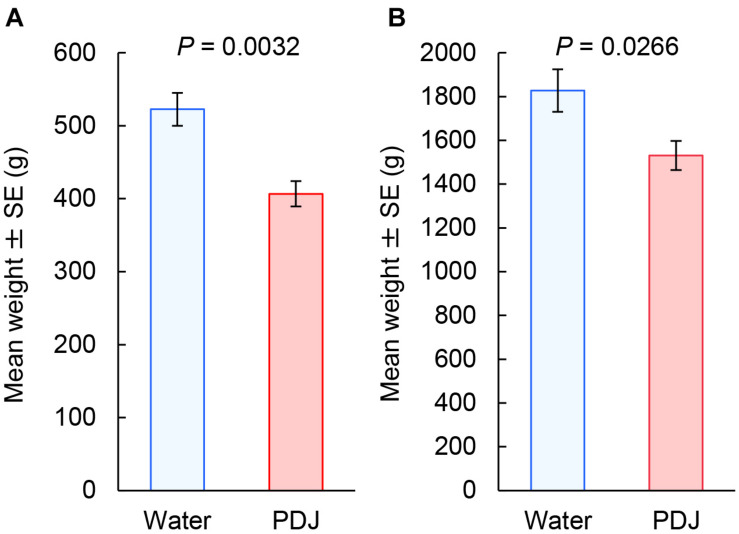
Weights (g) of both the aboveground **(A)** and belowground **(B)** parts of Japanese radish plants treated with PDJ and water (GLMM).

The weights of both the aboveground and belowground parts of the radish plants treated with PDJ were significantly lower than those of the control plants (aboveground parts: *P* = 0.0032; belowground parts: *P* = 0.027) (Figure 4).

### Analysis of the Headspace Volatiles of Japanese Radish Plants

Thirteen and 12 compounds were detected in the headspaces of the treated and control plants, respectively (Table 1). To detect compositional differences, we applied a PCoA. The first two axes (MDS1 and MDS2) explained 90.3% and 5.3% of the difference in volatile composition between the plants. The blend of volatiles from the control and treated plants were grouped in different areas with significant difference (PERMDISP: *P* = 0.1, PERMANOVA: *P* = 0.01, Supplementary Figure 3). The compounds that significantly contributed to the difference between the two groups were (*E*)-β-caryophyllene (72.5%, *P* = 0.01), DMNT (17.3%, *P* = 0.0091), (*E,E*)-α-farnesene (3.3%, *P* = 0.0091), α-humulene (3.0%, *P* = 0.0091), caryophyllene oxide (1.1%, *P* = 0.0091), α-copaene (0.8%, *P* = 0.0091), methyl salicylate (0.4%, *P* = 0.022), and germacrene D (0.2%, *P* = 0.047). The amounts of these compounds detected in the headspace of the treated plants were significantly higher than those of the control plants (*P* < 0.001, Table 1). The amounts of (*Z*)-3-hexen-1-yl acetate, 4-methylthio-3-butenyl isothiocyanate, menthol, and an unknown terpenoid were not significantly different between the PDJ-treated and control plants (Table 1).

**TABLE 1:** The volatiles emitted from PDJ-treated Japanese radish plants and those emitted from water-treated conspecific plants.

	Relative peak area (×10^–5^)	
Compounds	PDJ	Water control
(*Z*)-3-Hexen-1-yl acetate	144.7 ± 36.2	133.8 ± 38.0
Methyl salicylate	108.4 ± 19.1	9.1 ± 2.3 ***
4-Methylthio-3-butenyl isothiocyanate	45.2 ± 40.2	34.0 ± 19.9
Myrcene	41.9 ± 2.2	14.0 ± 2.1 ***
(*E*)-4,8-dimethyl-1,3,7-nonatriene	4387.0 ± 897.0	15.9 ± 2.5 ***
Menthol	107.9 ± 5.9	95.4 ± 4.6
α-Copaene	203.8 ± 9.4	10.5 ± 1.9 ***
Germacrene D	52.4 ± 3.2	6.1 ± 1.3 ***
(*E*)-β-Caryophyllene	17777.2 ± 1158.9	118.8 ± 24.9 ***
α-Humulene	731.6 ± 60.5	0.0 ± 0.0 ***
(*E,E*)-α-Farnesene	789.7 ± 67.1	5.4 ± 1.0 ***
Caryophyllene oxide	272.8 ± 25.0	12.2 ± 3.4 ***
Unknown terpenoid	219.7 ± 32.9	370.5 ± 116.7

Data are represented by mean ± standard error. Asterisks indicate that there is a significant difference in quantity between treatments. ***P < 0.001 (*t*-test).

### qRT-PCR Analyses

**FIGURE 5:**
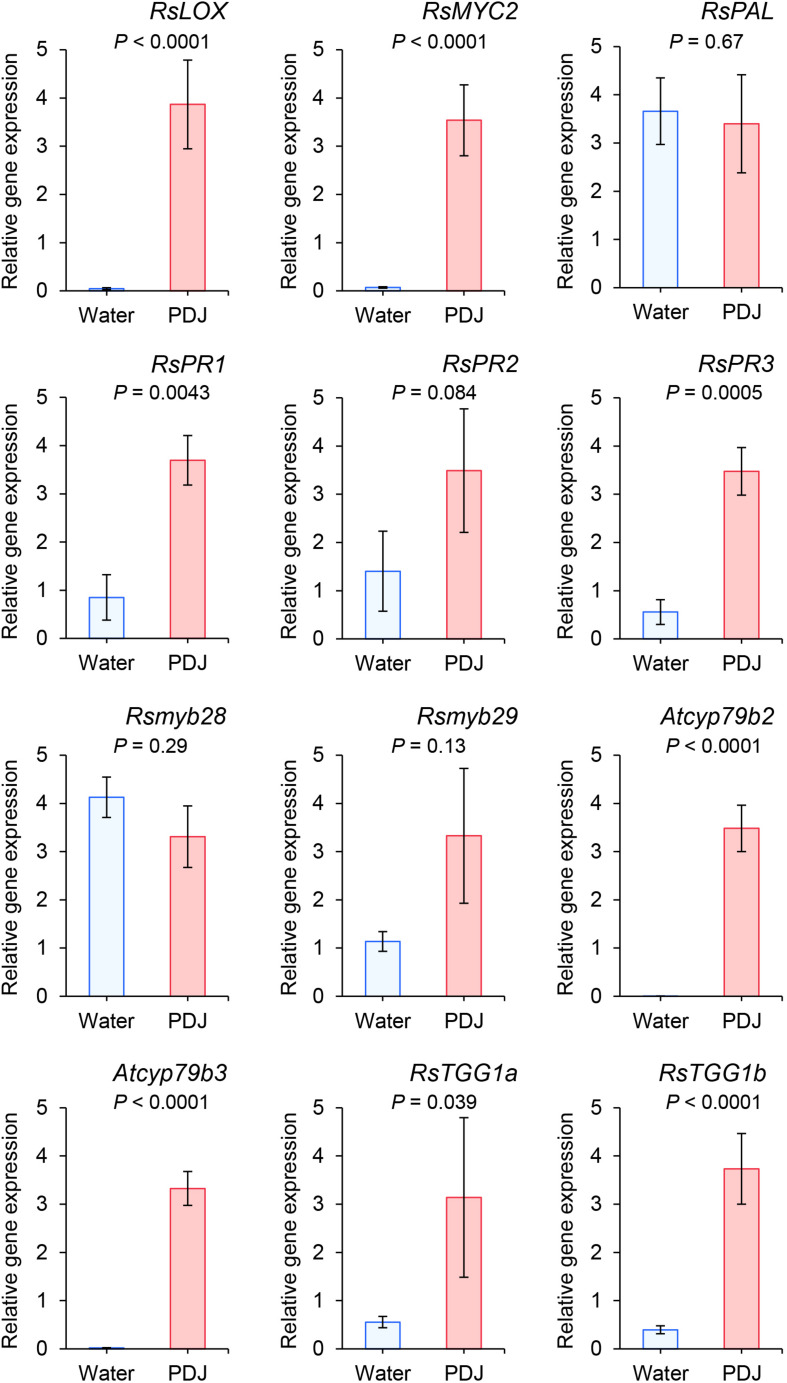
Expression levels of *RsLOX* and *RsMYC2*, which are regulated by the jasmonic acid signaling pathways, *RsPAL*, *RsPR1*, *RsPR2*, and *RsPR3*, which are regulated by the salicylic acid signaling pathways; *Rsmyb28* and *Rsmyb29*, orthologs of *Atmyb28* and *Atmyb29* which regulated aliphatic glucosinolate biosynthesis, *Rscyp79b2* and *Rscyp79b3*, orthologs of *Atcyp79b2* and *Atcyp79b3* which regulate indole glucosinolate biosynthesis; and *RsTGG1* and *RsTGG2*, orthologs of myrosinase genes, *AtTGG1* and *AtTGG2*. The number of replications for all genes was five, except for *RsPR1* (*n* = 4) for PDJ-treated Japanese radish plants (*t*-test).

We studied the effects of PDJ treatment on the expression levels of *RsLOX*, and *RsMYC2*, which are regulated by the JA signaling pathway, and *RsPAL, RsPR1*, *RsPR2, and RsPR3* which are regulated by the salicylic acid (SA) signaling pathway. The expression of *RsLOX*, *RsMYC2*, *RsPR1* and *RsPR3* was significantly upregulated and that of *RsPR2* was marginally significantly upregulated when the plants had been treated with PDJ (*RsLOX*: *P* < 0.0001; *RsMYC2*: *P* < 0.0001; *RsPR1*: *P* = 0.0043; *RsPR2*: *P* = 0.084; *RsPR3*: *P* = 0.0005). In contrast, the expression level of *RsPAL* was not significantly different after PDJ treatment (*P* = 0.69) (Figure 5).

We also studied the effects of PDJ treatment on the expression levels of *Rsmyb28* and *Rsmyb29*, the orthologs of *Atmyb28* and *Atmyb29* which regulate aliphatic glucosinolate biosynthesis, *Rscyp79b2* and *Rscyp79b3*, orthologs of *Atcyp79b2* and *Atcyp79b3* which regulate indole glucosinolate biosynthesis, and *RsTGG1* and *RsTGG2*, orthologs of myrosinase genes, *AtTGG1* and *AtTGG2*. The expression of *Rscyp79b2*, *Rscyp79b3*, *RsTGG1a*, and *RsTGG1b* was significantly upregulated when the plants had been treated with PDJ (*Rscyp79b2*: *P* < 0.0001; *Rscyp79b3*: *P* < 0.0001; *RsTGG1a*: *P* = 0.038 and *RsTGG1b*: *P* < 0.0001). In contrast, the expression levels of *Rsmyb28* and *Rsmyb29* were not significantly different after PDJ treatment (*Rsmyb28*: *P* = 0.29 and *Rsmyb29*: *P* = 0.13) (Figure 5).

## Discussion

Previous laboratory and greenhouse studies have shown that crops treated with PDJ become more resistant against herbivores directly or indirectly (see section “Introduction”) (Mandour et al., 2013; Uefune et al., 2014; Sobhy et al., 2015; Matsuura et al., 2020). These studies indicated that PDJ has the potential to increase the defense responses in non-infested plants of different classes (monocotyledons and dicotyledons) against herbivores with different feeding guilds (chewing pests: common armyworms; sucking pests: two-spotted spider mites). In the present study, we showed that PDJ treatment was effective in reducing the number of aphids, leaf-mining fly larvae, vegetable weevils, and thrips, without affecting the number of lepidopteran larvae, including those of *P. rapae* and few species of the subfamilies Hadeninae and Plusiinae, under open common garden conditions. Furthermore, PCoA analyses revealed that the community composition on the treated plants was significantly different from that on control plants in November and December, but not in October. In both November and December, aphids were the most important factor that explained the difference in the community composition. The other herbivores that influenced the composition were different between these 2 months: leaf miner fly larvae and thrips in November, and vegetable weevils, mummies, and cabbage butterflies in December.

The number of parasitized aphids (mummies) was significantly lower on the treated plants than on the control plants, which could be explained by the reduction in the number of host aphids. Poisson regression analyses revealed that significantly higher number of mummies were recorded on the treated plants when the number of aphids increased, suggesting that the treated plants attracted a higher number of aphid parasitoids than the control plants. PCoA analyses showed that the composition of volatiles from the treated plants and that from the control plants was significantly different, because the treatment also resulted in the production of PDJ-induced volatile compounds: one monoterpene (myrcene), six sesquiterpenes, one homoterpen (DMNT), and methyl salicylate. Some PDJ-induced volatiles from the treated plants probably attracted the parasitoid wasps (induced indirect defense). Among the induced volatiles, (*E*)-β-caryophyllene has been reported to attract *Aphidius ervi* Haliday, a parasitoid of several aphid species, including Brassica aphids (Sasso et al., 2007). However, *A. ervi* did not occur in the experimental area. Additionally, corn plants treated with PDJ have been reported to attract parasitic wasps (see section “Introduction”) (Mandour et al., 2013). The PDJ-induced compounds found in this study were not recorded in the headspace of Brussels sprouts sprayed with 1 mM JA solution except (*Z*)-3-hexen-1-yl acetate, a common green leaf volatile compound (Bruinsma et al., 2009). van Dam et al. (2010) identified biologically relevant compounds involved in the attraction of parasitoid wasps, *Cotesia glomerata*, in the headspace of feral *Brassica oleracea* plants that are treated with JA and host herbivory. They identified DMNT and six monoterpenes, the latter of which were not induced in this study, except myrcene. Qiu et al. (2012) reported that JA treatment to shoots of feral *B. oleracea* resulted in increased attraction of *Cotesia glomerata*, a parasitoid of cabbage white butterfly (*Pieris rapae*) larvae.

We did not detect the natural enemies of the leaf-mining fly larvae, vegetable weevils, thrips, and lepidopteran larvae (*P. rapae*, Hadeninae spp. and Plusiinae spp.). Thus, we could not evaluate the possible effects of PDJ on the indirect defenses against these herbivores.

JA and SA signaling pathways are antagonistic to each other (Yang et al., 2019). Under laboratory conditions, spraying PDJ on Japanese radish plants resulted in the induction of *LOX* and *MYC2* (regulated by the JA signaling pathways). Interestingly, we also observed an inconsistent induction of genes regulated by the SA signaling pathways; *PR1, PR2, and PR3* were induced, while *PAL* was not. In Arabidopsis, only the JA signaling pathway was induced in response to PDJ treatment (H. Abe, unpublished data). Clarifying this inconsistency, and the molecular mechanisms involved in the difference between Japanese radish plants and Arabidopsis are interesting. Papadopoulou et al. (2018) showed that *PR1* in *Brassica rapa* shoots was a unique marker gene for the SA pathway, while *B. rapa* roots *PR1* was induced not only by SA treatment, but also by the treatment of ethephon that did not affect SA levels. Thus, the widely used marker genes may not show specific responsiveness to single hormone applications in Japanese radish plants.

Induction of JA and SA signaling pathways results in the induction of direct defense responses in plants (Smith et al., 2009). Here, spraying the 100 times-diluted commercial formulation (5%) of PDJ on Japanese radish plants differentially activates their JA and SA signaling pathways. The induction of these pathways would in part explain the reduction of insect pests either directly or indirectly, except the lepidopteran larvae. In this study, as Japanese radish plants were cultivated for commercial purpose, we were not allowed to sample leaves to analyze JA and/or SA-inducible secondary compounds that might confer direct defense against aphids, thrips, vegetable weevils, and leaf-mining fly larvae during the experiments.

We also observed the effects of the PDJ treatments on the expression of the genes related to glucosinolate biosynthesis. The increase of *Rscyp79b2*, *Rscyp79b3*, *RsTGG1a*, and *RsTGG1b* expression in the treated plants indicated the induction of indole glucosinolate biosynthesis and the subsequent isothiocyanate formation. Whether these inductions affected the community composition on the treated plants remained unanswered. Both aliphatic and indole glucosinolates make plants more susceptible to attack by specialist herbivores, *P. rapae* and *Plutella xylostella* (Müller et al., 2010). Kim et al. (2008) reported that the post-ingestive breakdown of indole glucosinolates provides a defense against herbivores such as aphids.

Induced defenses are suggested to have evolved to save costs in the absence of herbivores (Agrawal et al., 1999). This mean, trade-offs between induced defense and plant growth are expected. In agricultural crops, these trade-offs may not be found, due to the addition of nutrients to the system and alleviation of competition (e.g., Siemens et al., 2002; Backmann et al., 2019). In contrast, we found that both the weights of the aboveground and belowground parts of the treated Japanese radish plants were significantly lower than those of the control plants, even though the PDJ-treated plants suffered less herbivory by aphids, leaf-mining fly larvae, and thrips than the control plants. This indicates that also in agricultural setting the costs of induction may outweigh the benefits. Alternatively, the direct effects of PDJ might explain the results. The concentration of PDJ used in this study was 5–20 times higher than that of what farmers used. This may have triggered a stronger response in PDJ- treated Japanese radish plants than those grown under normal conditions, thus leading to growth inhibition. Matsuura et al. (2020) reported that spraying PDJ on greenhouse-grown tomato slightly inhibited their initial growth but the fruit yield and quality were not affected. Azis et al. (2020) reported that no significant suppressive effect of PDJ was observed in the aerial parts of komatsuna plants (*Brassica rapa* var. periviridis) and eggplant (*Solanum melongena* L.), but a significant inhibitory effect was found in the roots of both the plant species when treated with PDJ at certain concentrations. Furthermore, in komatsuna, a lower concentration of PDJ resulted in an increase in root weight (Azis et al., 2020).

In conclusion, this study revealed a novel function of PDJ in pest control under open common garden conditions. Spraying the 100 times-diluted commercial formulation (5%) of PDJ every week reduced the incidences of aphids, leaf-mining fly larvae, vegetable weevils, and thrips; however, the incidences of lepidopteran larvae (*P. rapae* Hadeninae spp. and Plusiinae spp.) were not affected by the treatment. Further, the weights of the aboveground and belowground parts of treated plants were significantly lower than those of the control plants. These positive and negative effects might depend on the dose of PDJ as reported by Azis et al. (2020). Therefore, further investigations on the effects of treating Japanese radish plants, as well as other crops (see section “Introduction”), with different concentrations of the formulated PDJ on their growth and defenses against herbivores are required.

## Data Availability Statement

The datasets for this study are included in the article/Supplementary Material.

## Author Contributions

KeY, MU, HA, RO, and YO designed and conducted the experiments and analyzed the data. KiY and JT analyzed the data. JT, MU, and HA wrote the manuscript. All authors have approved the final manuscript for publication.

## Conflict of Interest

The authors declare that the research was conducted in the absence of any commercial or financial relationships that could be construed as a potential conflict of interest.

## Publisher’s Note

All claims expressed in this article are solely those of the authors and do not necessarily represent those of their affiliated organizations, or those of the publisher, the editors and the reviewers. Any product that may be evaluated in this article, or claim that may be made by its manufacturer, is not guaranteed or endorsed by the publisher.
