# Supplementary figures and images for: Effects of Prohydrojasmon on the Number of Infesting Herbivores and Biomass of Field-Grown Japanese Radish Plants
Source: Front Plant Sci. 2021 Aug 12;12:695701. doi: 10.3389/fpls.2021.695701 (PMC8406856; doi:10.3389/fpls.2021.695701)

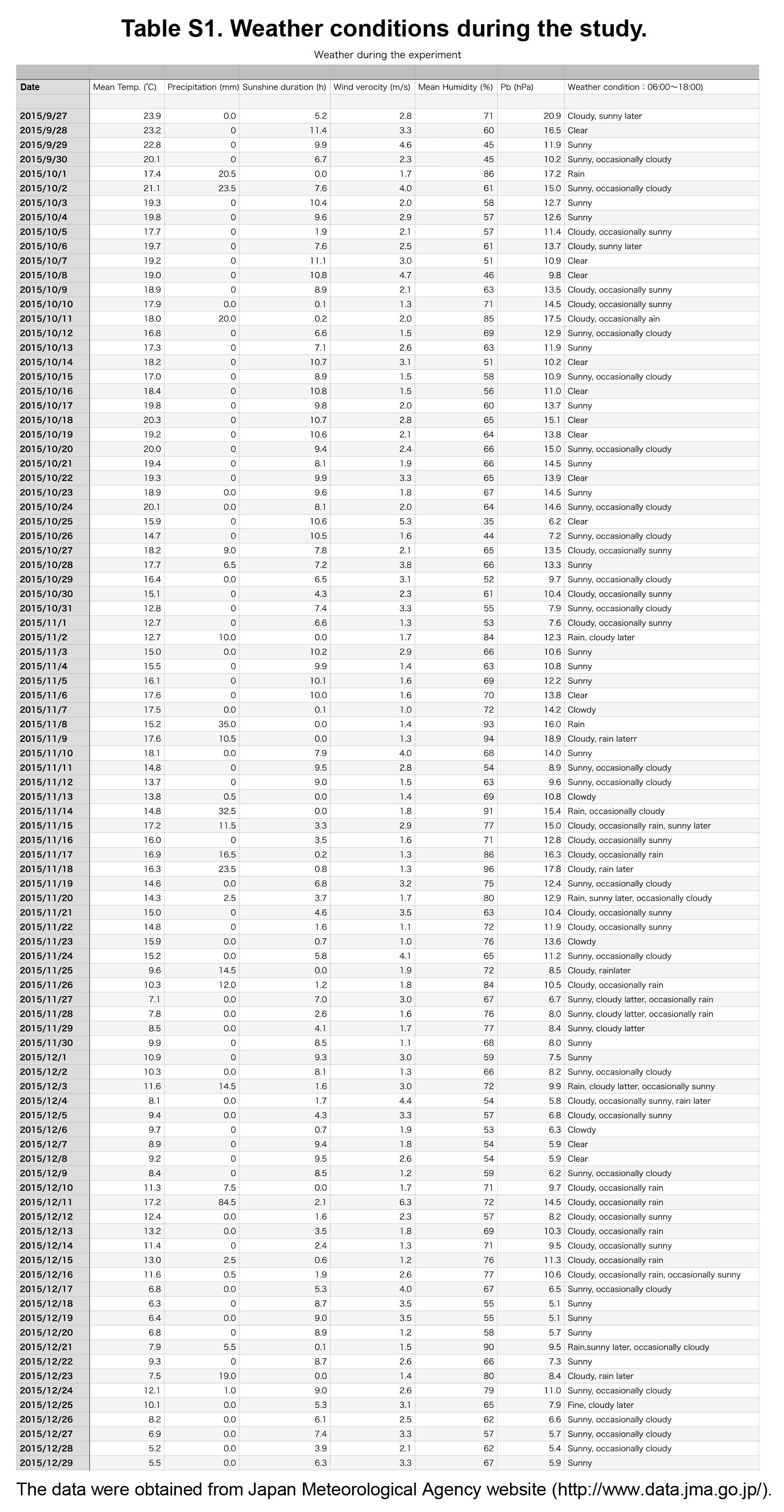

Supplement: Supplementary file 2 [file Image_1.JPEG]

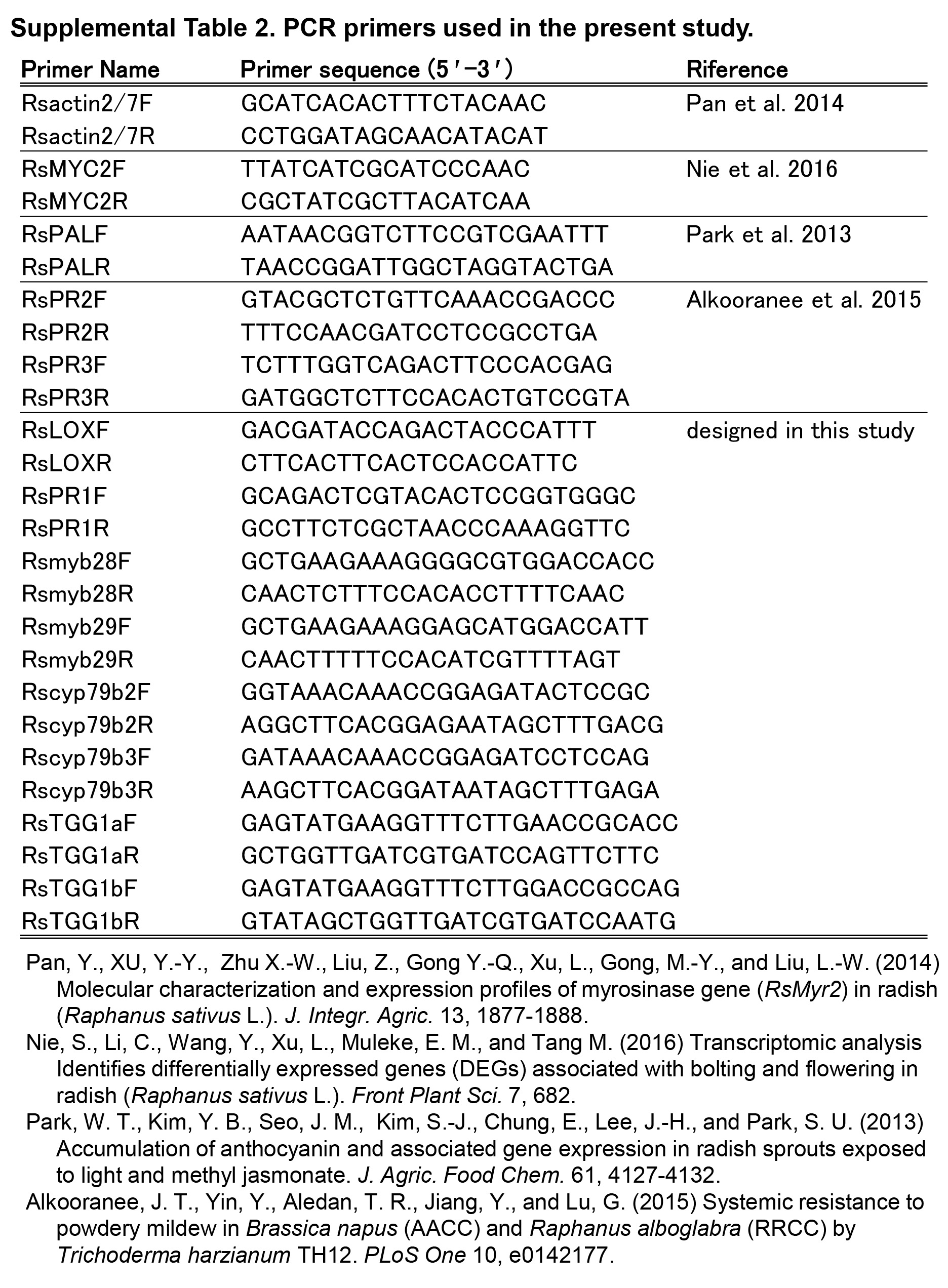

Supplement: Supplementary file 3 [file Image_2.JPEG]

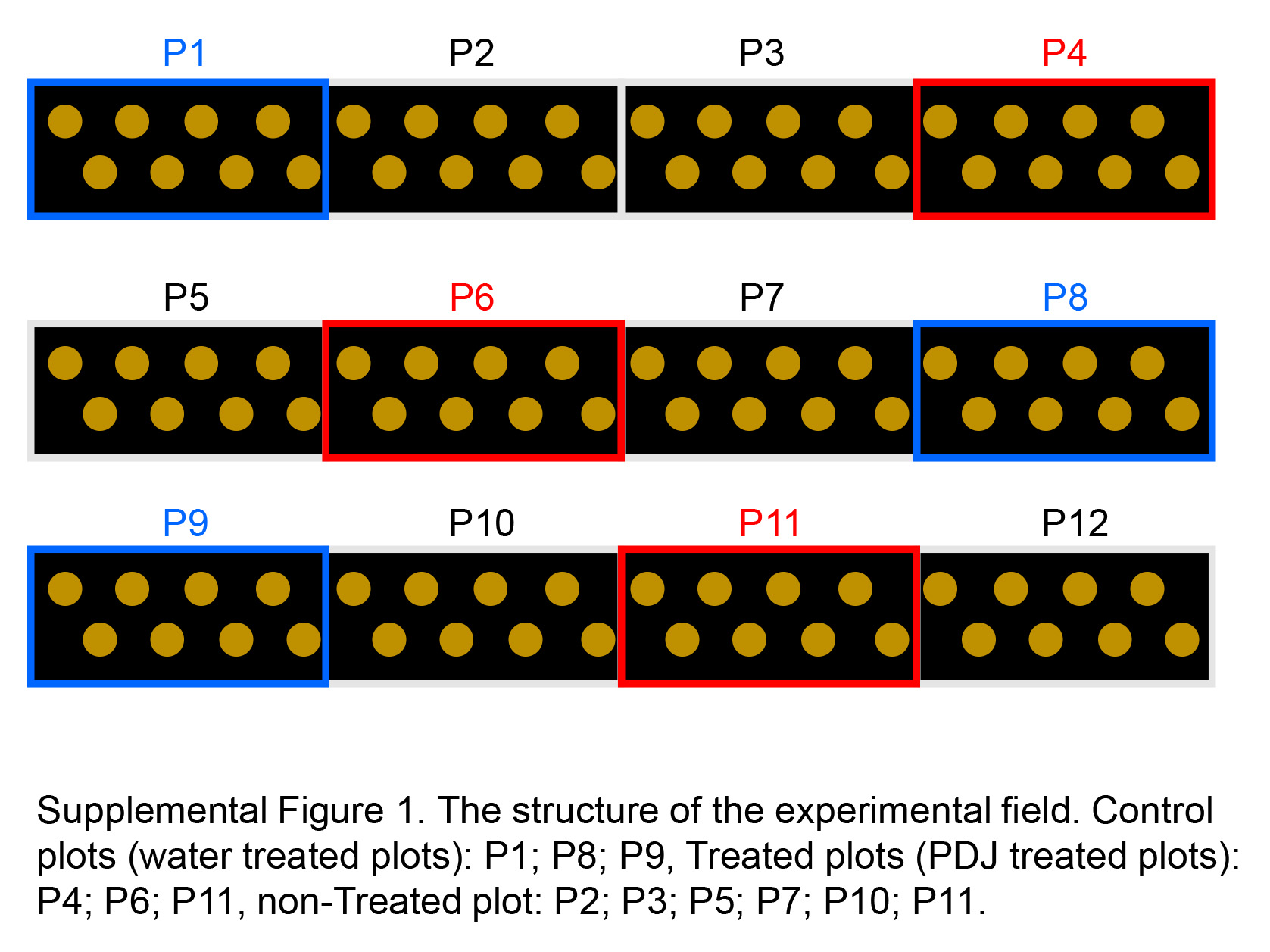

Supplement: Supplementary file 4 [file Image_3.JPEG]

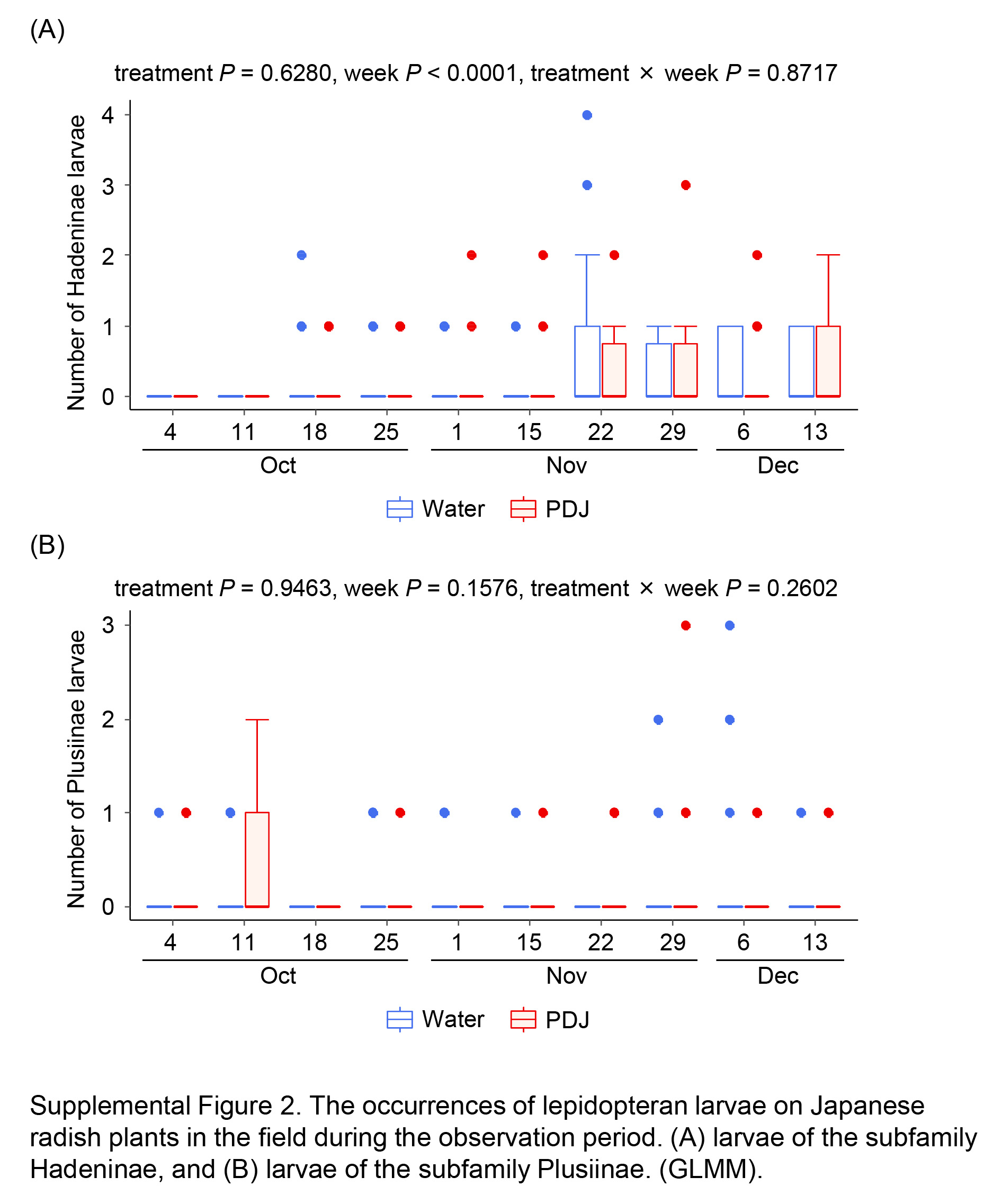

Supplement: Supplementary file 5 [file Image_4.JPEG]

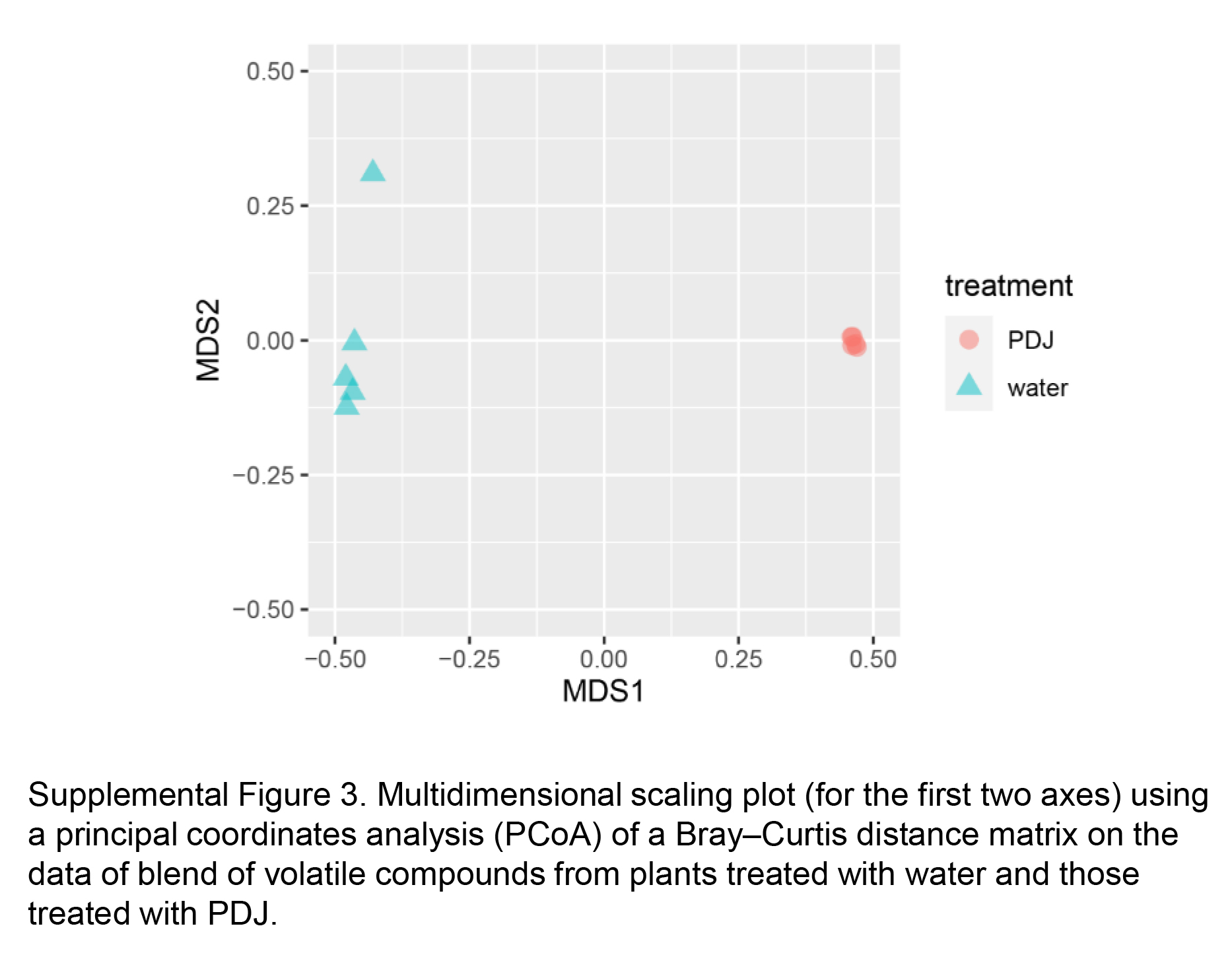

Supplement: Supplementary file 6 [file Image_5.JPEG]
